# Assay Reproducibility in Clinical Studies of Plasma miRNA

**DOI:** 10.1371/journal.pone.0121948

**Published:** 2015-04-08

**Authors:** Jonathan Rice, Henry Roberts, James Burton, Jianmin Pan, Vanessa States, Shesh N. Rai, Susan Galandiuk

**Affiliations:** 1 Price Institute of Surgical Research, Hiram C. Polk Jr., M.D. Department of Surgery, University of Louisville School of Medicine, Louisville, KY, United States of America; 2 Department of Bioinformatics and Biostatistics, University of Louisville School of Public Health and Information Sciences, Louisville, KY, United States of America; Deutsches Krebsforschungszentrum, GERMANY

## Abstract

There are increasing reports of plasma miRNAs as biomarkers of human disease but few standards in methodologic reporting, leading to inconsistent data. We systematically reviewed plasma miRNA studies published between July 2013-June 2014 to assess methodology. Six parameters were investigated: time to plasma extraction, methods of RNA extraction, type of miRNA, quantification, cycle threshold (Ct) setting, and methods of statistical analysis. We compared these data with a proposed standard methodologic technique. Beginning with initial screening for 380 miRNAs using microfluidic array technology and validation in an additional cohort of patients, we compared 11 miRNAs that exhibited differential expression between 16 patients with benign colorectal neoplasms (advanced adenomas) and 16 patients without any neoplasm (controls). Plasma was isolated immediately, 12, 24, 48, or 72 h following phlebotomy. miRNA was extracted using *two different* techniques (Trizol LS with pre-amplification or modified miRNeasy). We performed Taqman-based RT-PCR assays for the 11 miRNAs with subsequent analyses using a variable Ct setting or a fixed Ct set at 0.01, 0.03, 0.05, or 0.5. Assays were performed in duplicate by *two different* operators. RNU6 was the internal reference. Systematic review yielded 74 manuscripts meeting inclusion criteria. One manuscript (1.4%) documented all 6 methodological parameters, while < 5% of studies listed Ct setting. In our proposed standard technique, plasma extraction ≤12 h provided consistent ΔCt. miRNeasy extraction yielded higher miRNA concentrations and fewer non-expressed miRNAs compared to Trizol LS (1/704 miRNAs [0.14%] *vs* 109/704 miRNAs [15%], not expressed, respectively). A fixed Ct bar setting of 0.03 yielded the most reproducible data, provided that <10% miRNA were non-expressed. There was no significant intra-operator variability. There was significant inter-operator variation using Trizol LS extraction, while this was negligible using modified miRNeasy. For standardized reporting, we recommend plasma extraction ≤ 12 h, using modified miRNeasy extraction and utilizing a 0.03 Ct.

## Introduction

MicroRNAs are small 19–23 nucleotide noncoding ribonucleic acids (RNA) that bind to complementary sequences on the 3' untranslated region of target messenger RNAs (mRNA) [1]. Consequently, microRNAs (miRNA) post-transcriptionally regulate mRNA expression and are essential in numerous molecular regulatory pathways [2]. miRNA expression profiles have been shown to be unique to both the source material (i.e. plasma, tissue, etc.) and the disease process being investigated. miRNA profiles have, therefore, emerged as prospective biomarkers for cancer and many other human diseases [3–7].

This has led to a rapid proliferation of miRNA research. Unfortunately, many studies have been conducted without attention to standardization of methods or reproducibility of results, particularly with respect to studies of plasma miRNA. In many reports, it is difficult to deduce the actual methods used for analysis. This has led to the use of different extraction protocols, and various methods of quantification and statistical analysis, which, in turn, are a source of variability (Table 1). In part, due to this lack of standardization, many different miRNAs have been reported to be associated with a given disease process [5]. There is ongoing controversy over the optimal analytic methods for studies of miRNA in plasma[8].

**Table 1:** Multiple sources of variability in microRNA data and literature search [9–84].

Parameter		Source of Variability
**Biologic Sample**	Tissue	**Preservation Method**-Formalin fixed-paraffin embedded-Snap Frozen **Collection Method**-Laser caption microdissection-Macrodissection (includes stroma)
	Plasma	- Collection method (e.g. EDTA tube)-Method to isolate plasma
	Other body fluids (urine, cerebrospinal fluid, etc.)	- Collection method
**RNA Extraction**	Time to extraction* †	- Immediate (within 24 h)-Delayed
	Method of RNA extraction* †	- Guanidinium thiocyanate-phenol chloroform based (e.g. Trizol LS, LifeTechnologies) -Glass fiber filter-based methods (e.g. miRVANA, Ambion)-Phenol/guanidine-based with and silica membrane based purification (e.g. miRNeasy, Qiagen)
	Type of miRNA*	- Total -Fractional (e.g. exosomal)
**miRNA Detection & Statistical Analysis**	miRNA characterization	- Cyanine dye-based RT qPCR (SYBR Green) detection-Fluorogenic 5’ nuclease-based RT qPCR detection-Deep sequencing
	Quantification*	- Absolute (addition of exogenous miRNA e.g. *C. elegans* miRNA)-Relative (use of an internal reference e.g. RNU48, RNU44, RNU47, RNU6, miR-16)
	Cycle threshold bar* †	- Fixed threshold-Variable threshold
	Statistical analysis* †	- Paired t-Test-Wilcoxon Signed Rank test-CT ratios-Rank-based
	Reproducibility†	- Intra-operator comparisons-Inter-operator comparisons

*Items investigated in systematic literature review.

^†^ Items investigated in our proposed standard methodologic technique.

Since the discovery of miRNAs, their detection in blood has received much attention due to the ease of access and ready availability of peripheral blood as compared to tissue [5]. Initially, we performed a systematic review of publications focusing on plasma miRNA in order to ascertain what methods and reporting criteria were currently being utilized. We then we used a panel of 11 selected miRNA to study the effect of 5 of the variables shown in Table 1 on data obtained in plasma miRNA studies, namely the effect of:

- Time to plasma extraction
- Method of RNA extraction
- Cycle threshold bar setting
- Intra-operator variability
- Inter-operator variability

## Materials and Methods

### Systematic Review

In order to determine the consistency and current status of methods reporting of clinical studies of plasma miRNA, we retrieved original manuscripts published from July 1, 2013, until June 30, 2014. We utilized a single search engine (PubMed) without language restriction using the following search words: plasma, microRNA, and human. We excluded review articles, case reports, or non-English language articles. Remaining articles were then obtained for review. These were then graded as to how many of the following criteria were clearly documented in the *Materials and Methods* sections: 1) time of plasma extraction, 2) method of RNA extraction, 3) type of miRNA used (total *vs* exosomal), 4) method of quantification (external vs internal reference), 5) cycle threshold bar setting, and 6) methods of statistical analysis (items denoted by * in Table 1).

### microRNA Selection

An eleven miRNA panel with RNU6 as an internal reference was used in this study. These miRNAs were selected based upon unpublished data from our laboratory utilizing 380 miRNA arrays (Applied Biosystems, Carlsbad, California) to determine miRNA expression in the plasma of 20 colorectal cancer (CRC) patients, and 10 patients each with colorectal advanced adenoma (CAA)(adenomatous polyps> 0.6 cm in maximal diameter), breast cancer (BC), lung cancer (LC), pancreatic cancer (PC), and 10 controls.

In order to determine sample size, the most important aspect is adjustment of the significance level (alpha). For screening studies, we use Jung’s procedure to adjust the alpha. Using the method of Jung to find about 5% of features to be significant at a false detection rate (FDR) of 5%, the adjusted alpha will be 0.0038. With any two groups, a minimum of n_1_ = 10 and n_2_ = 10 using a two sample t test, we can detect at least 2.7 fold means (which we have observed in our preliminary data sets) using the common standard deviation at significance level of 0.0038 and power of 80%.

**Fig 1:**
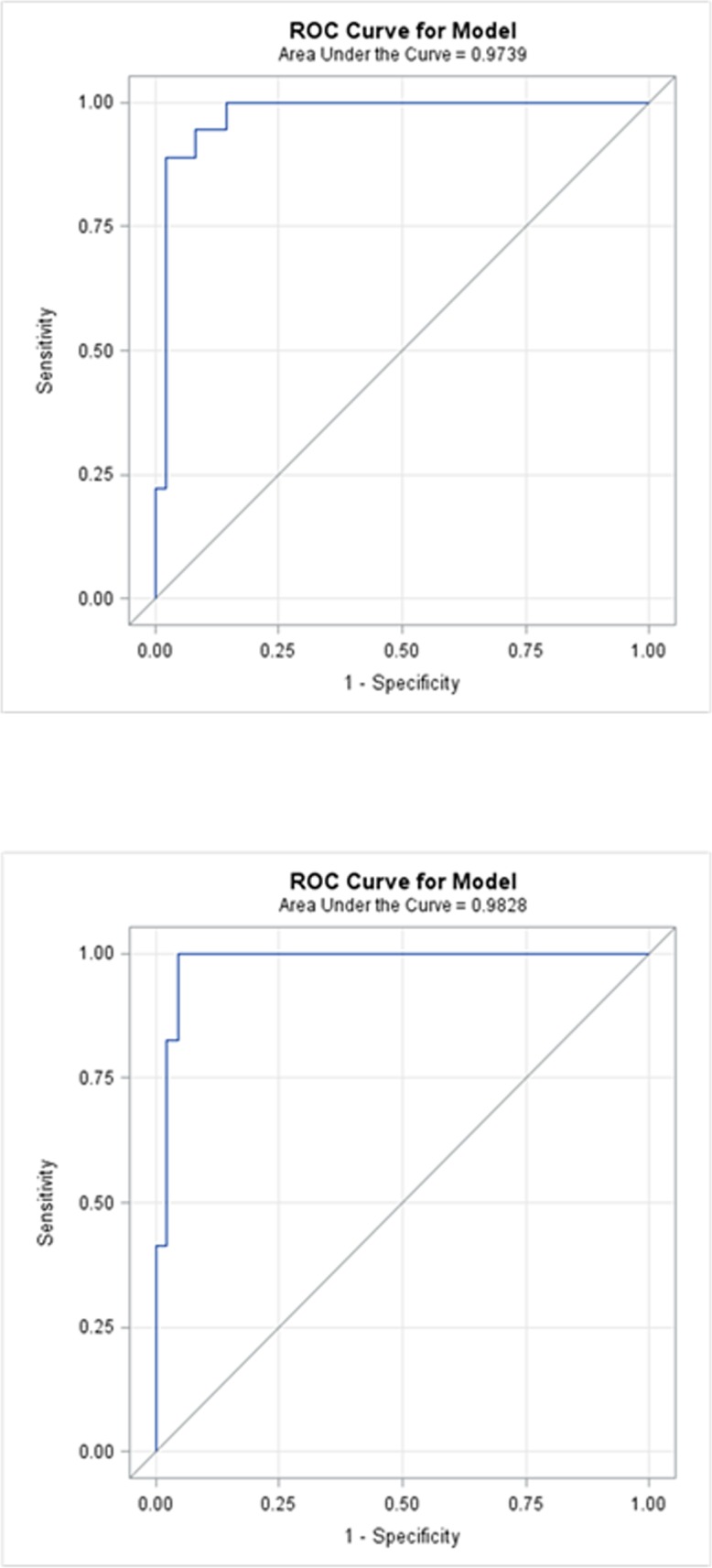
A) Receiver operator characteristic (ROC) curve for miR-523,miR-218,miR-142-3p,miR 27a,miR-21. Colorectal cancer (n = 20) vs. Breast cancer +Pancreatic cancer + Lung cancer (n = 30 [10 each group]). B) ROC Curve for miR-523, miR-218, miR-142-3p,miR-27a,miR-376c,miR-374. Colorectal cancer (n = 20) + Colorectal adenoma (n = 10) vs. Breast cancer +Pancreatic cancer + Lung cancer (n = 30 [10 each group]).

With respect to choice of number of miRNA in our panel, it was our expectation that no more than 10% of miRNA would be differentially expressed between cases and controls after adjusting the p values for multiple comparisons. Of these, in turn, one would not expect more that 0.5 to 3% of miRNA to be able to accurately identify cases and controls. Ten miRNA and one reference (housekeeping gene) miRNA were, therefore, chosen (approximately 3%). Statistical analysis using ANOVA identified 11 significantly dysregulated miRNAs specific for colorectal neoplasia (Table 2). Multiple test control was based on controlling the false discovery rate (FDR) at 10%. A logistic regression model was established using the top up-regulated miRNAs and used for predicting the adenoma and control groups for the validation data. The sensitivity and specificity for this prediction were calculated. The receiver operator characteristic (ROC) curves with AUC values were microRNA-rated using current versions of SAS [85] and R [86–88] (Fig 1a & 1b). These data are not the focus of this report; however, these selected miRNA were utilized for the present study to evaluate the effect of time to plasma extraction, the effect of multiple samples drawn from an individual over time, the method of RNA extraction, cycle threshold bar setting, and intra- as well as inter-operator variability.

**Table 2:** Results of ANOVA analysis for initial microRNA selection.

Two-Way ANOVA: CRC (n = 20) vs. BC+PC+LC (n = 30)		
microRNA	Raw p-value	Fold Change
miR-374	< 0.0001	28.5
miR-142-3p	< 0.0001	23.3
miR-523	< 0.0001	8.8
miR-374-5p	0.0001	90.2
miR-376c	0.001	13.2
miR-27a	0.001	10.3
miR-21	0.016	11.2
**Two-Way ANOVA: CAA (n = 10) vs. BC+PC+LC+CRC (n = 50)**		
**microRNA**	**Raw p-value**	**Fold Change**
miR-520d-5p	0.001	7.9
miR-122	0.002	2.5
miR-485-3p	0.009	10.2
miR-218	0.024	1.8

Abbreviations: CRC, colorectal cancer; BC, breast cancer; PC, pancreatic cancer; LC, lung cancer; CAA, colorectal advanced adenoma.

### Patient Population

The University of Louisville Institutional Review Board reviewed and approved this study. Written informed consent was obtained from all subjects who were treated at a single university-based colorectal surgery practice. The patient population consisted of 16 patients with colorectal advanced adenomas, and 16 patients without colorectal neoplasia (controls). The patient groups were age-, race-, and gender-matched. Prior to patient treatment, 6 mL of peripheral whole blood was obtained in EDTA tubes (Becton-Dickinson, Franklin Lakes, NJ) via venipuncture from the adenoma group and from individuals in the “control” group at the time of routine screening colonoscopy. The latter group of individuals (n = 16) had no colonic neoplasia or inflammatory bowel disease. Blood was stored at 4°C until plasma isolation. Plasma was isolated within 24 h of venipuncture, unless noted otherwise (see section below “*Time to Plasma Extraction*”). Patient demographics are displayed in Table 3 and did not differ between patient groups.

**Table 3:** Patient demographics.

	Colorectal Advanced Adenomas (n = 16)	Patients Without Colorectal Neoplasia or IBD (n = 16)
**Race**		
Caucasians	16	16
**Age**		
Mean (± standard deviation) years	65±9.3	65±9.2
Median (range) years	64 (49–85)	64 (49–85)
**Gender**		
Male	8	8
Female	8	8
**Size of Adenoma**		
Mean (range) cm	2.2 (0.8–5.5)	Not Applicable

Abbreviations: IBD, inflammatory bowel disease.

### Time to Plasma Extraction

Five 6 mL aliquots of peripheral blood from 6 controls were obtained and stored at 4°C until extraction. Plasma was extracted at different time points: immediately (within 30 minutes after phlebotomy) and then at 12, 24, 48, and 72 h post-phlebotomy. Whole blood was centrifuged at 600 relative centrifugal force (rcf) for 15 minutes in order to isolate the plasma, which was then stored at -80°C for later use. Once plasma was isolated, it underwent downstream processing via the modified phenol/guanidine-based lysis and silica membrane-based extraction technique (miRNeasy, Qiagen, Venlo, Limburg).

### Effect of Repeated Sample Acquisition

In order to determine whether there was a difference between samples when plasma miRNA were analyzed in samples drawn at different times from the same individual, 6 mL peripheral blood was drawn from each of 12 healthy subjects without any neoplasia or inflammatory condition at 6:30 AM and 12 hours later at 6:30 PM on the same day. Plasma was extracted within 30 minutes of phlebotomy, and stored at -80°C for later use. Downstream processing was performed using the modified phenol/guanidine-based lysis and silica membrane-based extraction technique (miRNeasy, Qiagen, Venlo, Limburg) and methodology described below.

### Method of RNA Extraction

Total RNA was extracted from 250 μL plasma samples using either the Trizol LS reagent protocol (Ambion, Austin, Texas), which was modified by addition of an extended overnight drying period, or by a modified miRNeasy (Qiagen, Venlo, Limburg) extraction technique with yeast carrier [89, 90]. Total RNA purity was assessed using a Nanodrop 2000 spectrophotometer (Thermo Scientific, Middlesex, MA).

### Pre-Amplification

When using the TRIzol LS total RNA extraction technique, pre-amplification was necessary, as the majority of samples yielded quantities of total RNA < 500 ng. In our experience, this is the minimum amount needed for adequate expression and amplification in qPCR. The Applied Biosystems protocol for producing custom reverse transcription and pre-amplification pools with TaqMan miRNA assays was followed [91].

### Reverse Transcription

For reverse transcription, 10 ng of RNA were converted into cDNA utilizing the TaqMan miRNA reverse transcription kit with our custom 5X microRNA-specific stem-loop primer pool. The reverse transcription product was then added to the pre-amplification master mix and our custom 20X miRNA-specific stem-loop primer pool.

### MicroRNA Quantification

A Step-One Plus RT-PCR System (Life Technologies, Carlsbad, California), with default fast thermal cycling conditions, was used for RT-PCR. Individual Taqman probes (20x) were used during RT-PCR to bind to a complementary sequence in the target cDNA. These values were, in turn, normalized to the expression of the endogenous miRNA RNU6 as internal reference to calculate ΔC_t_ values.

### Setting of Cycle Threshold

**Fig 2:**
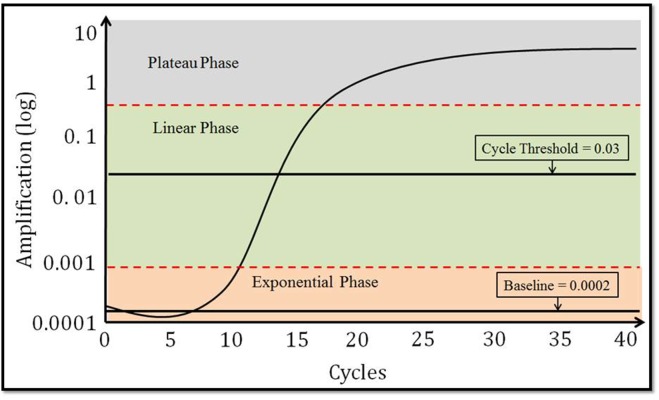
The phases and components of a PCR curves: (1) baseline, (2) exponential phase, (3) linear phase, (4) plateau phase, and (5) cycle threshold.

In order to compare cycle threshold (C_t_) values across plates, both fixed and variable thresholds were utilized. Since different miRNAs on the same plate may have different linear phases, a fixed threshold may not intersect across the linear phase. For this reason, the variable threshold is the default setting. Cycle threshold is illustrated in Fig 2, which shows the phases of the PCR curves: (1) the baseline, (2) the exponential phase, (3) the linear phase, and 4) the plateau phase. The RQ manager from Applied Biosystems may use a different threshold (variable) within the same plate if one selects the option “automatic C_t_”. Different thresholds will be chosen for different miRNAs according to the linear phases. However, these C_t_ values cannot be compared directly between different plates. The threshold needs to be considered in the analysis and adjustment of the C_t_ values is needed. In addition to this variable threshold setting, we examined the effect of fixed threshold settings of 0.01, 0.03, 0.05, or 0.5. Ct values, threshold, and 40 cycles fluorescence intensities are miRNA-rated from RQ manager 1.2, Applied Biosystems.

### Intra-operator Variability

Assays for individual miRNA for each patient sample were run in duplicate to permit assessment of intra-operator variability (comparison of duplicate samples).

### Inter-operator Variability

In order to assess inter-operator variation following total RNA extraction, two experienced operators separately performed subsequent sample processing, including reverse transcription, preamplification (for samples extracted using TRIzol LS), and qPCR. Each operator used the same thermocycler (Mastercycler, Eppendorf, Hamburg, Germany) and Step-One Plus RT-PCR System, but at different times on different days. The cycle threshold (C_t_) values were exported with the C_t_ bar set at 0.03.

### Statistical Methods

A portion of the statistical analysis plan is already discussed in the methods under the section “*miRNA selection*.” Given the clinical information, in addition to gene expression data, we use a full statistical model-based approach, such as ANOVA or ANCOVA to analyze the data. Another alternative is the use of a simple approach for comparing means or medians using a two-sample t test based on a parametric approach or a Wilcoxon rank sum non-parametric test [92, 93].

For data with a variable threshold, we considered the threshold as a covariate in the ANOVA model or used it in the normalization. The ANOVA model was fitted as follows.

$$▽{\text{C}}_{\text{t}}{\text{ = α + β}}_{\text{1}}{\text{ Group+ β}}_{2}{\text{ Operator+ β}}_{3}\text{ Group}\times {\text{Operator+ β}}_{\text{4}}{\text{log}}_{\text{2}}{\text{(T}}_{\text{t}}{\text{/T}}_{\text{R}}\text{)+ε}$$

where ▽C_t_ = C_t—_C_R_ for a fixed threshold or a variable threshold with a covariate log_2_(T_t_/T_R_), and ▽C_t_ = C_t—_C_R_—log_2_(T_t_/T_R_) for variable threshold using threshold in the normalization. ▽*C*_*t*_ = *C*_*t*_ *—C*_*R*_ for a fixed threshold or a variable threshold with a covariate log_2_ (T_t_ / T_R_), and ▽*C*_*t*_ = *C*_*t*_—*C*_*R*_—log_2_ (T_t_ / T_R_) for variable threshold using threshold in the normalization. T_t_ is the threshold intensity of the miRNA, C_R_ and T_R_ are the C_t_ values and the threshold intensity of reference gene *U*_6_, respectively. The error term Ɛ in the model is due to multiple factors (subject variability, operator/machine variability). We considered six ANOVA models, with the operator as a covariate or not, with interaction, Group×Operator, or not, with Operator = 1 or 2 only, and with the average of Operator = 1 and 2. Differences were considered significant for p-values < 0.05. To study the effect of threshold intensity, we applied a variable threshold and a wide range (0.01, 0.03, 0.05, or 0.5) of threshold settings. If the fixed threshold setting is used, the penultimate correction is not required. These data results are discussed in detail elsewhere (Rai et al., unpublished data, 2014).

Another critical approach is to build a prediction model to identify groups of individuals (such as cases and controls). Unlike high-throughput data analysis with tens of thousands of biomarker (genes), which involves hierarchical modeling, principal component analysis, heat map, and other methods, with a lesser number of biomarkers (miRNAs), we apply an ANOVA/ANCOVA model for model fitting and a logistic model for prediction [94, 95]. The classification method based on a logistic model is simple and is extensively used [88, 96, 97]. All statistical analyses were performed using SAS 9.3 and R [85] [86–88].

## Results

### Literature Review

**Fig 3:**
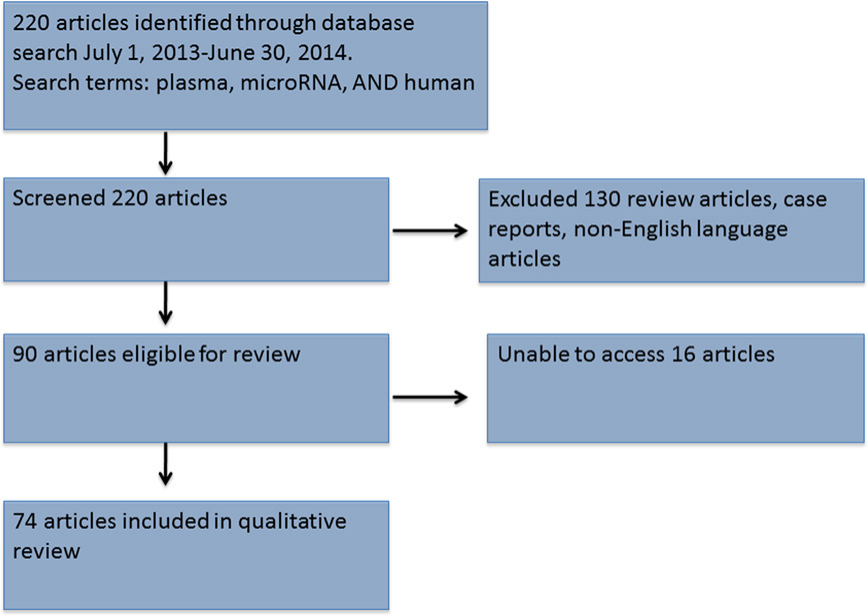
A PRISMA flow diagram illustrating the search strategy used[98].

Of the 220 retrieved abstracts, 130 were excluded because they were reviews, case reports, or non-English language manuscripts. Sixteen publications were unobtainable through our library, leaving 74 manuscripts available for review. A PRISMA flow diagram is shown in Fig 3 and data shown in Table 4 [98]. Although nearly one-third of studies did not mention time to plasma, in the vast majority (nearly 58%), plasma extraction was completed ≤2h after phlebotomy. Most authors used either Trizol, miRNeasy, or mirVana protocols for RNA extraction, and nearly all publications used total miRNA rather than exosomal miRNA. Equal proportions of manuscripts used internal and external references (43% and 42%, respectively). The most commonly utilized internal reference miRNAs were miR-16 (12 publications) and RNU-6 (7 publications). Seventy-one of the 74 reviewed publications did not describe the setting of the cycle threshold bar. All but 2 of the 74 (97%) papers stated at least some aspect of their statistical methods, with many using multiple, different testing methods as shown in Table 4. Among reviewed manuscripts, only 1 (1.4%) listed all 6 assessed criteria [9–84].

**Table 4:** Systematic review: 74 plasma miRNA publications July 1, 2013—June 30, 2014.

Methodologic Parameter Assessed	Number of studies (% of total 74 manuscripts reviewed)
**Time of Plasma Extraction**	
≤ 2h	44 (59.5)
>2h	8 (10.8)
Not Stated	22 (29.7)
**Method RNA Extraction** ^+^	
Trizol	13 (17.6)
miRNeasy	25 (33.8)
mirVana	30 (40.5)
Not Stated/Other	8 (10.8)
**Type of miRNA used**	
Total miRNA	72 (97.3)
Exosomal miRNA	2 (2.7)
**Cycle Threshold Bar Setting**	
Fixed	2 (2.7)
Variable	1 (1.4)
Not Listed	71 (95.9)
**miRNA Quantification** *	
External reference	31 (41.9)
Internal reference	32 (43.2)
Not specified	12 (16.2)
**Statistical Analysis Described** ^ψ^	74 (100)
t-test	30 (40.5)
Mann-Whitney	29 (39.2)
Receiver operating characteristic (ROC) curves	24 (32.4)
Wilcoxon	16 (21.6)
ANOVA	12 (16.2)
Other	14 (18.9)

^+^ Some manuscripts utilized more than one method of RNA extraction.

* Some manuscripts used both an internal and external reference.

^ψ^ Many manuscripts used multiple methods of statistical analysis.

### Time to Plasma Extraction

Our proposed standard technique provided a comparison. When performing plasma extraction from whole blood at different time points, we found that 6 miRNAs showed no differences in ΔCT values. In contrast, 5 miRNAs expressed a statistically significant difference in ΔCT values between immediate plasma extraction and extraction ≥ 24 h for 1 miRNA (miR-122), between immediate extraction and extraction after ≥48 h for 2 miRNAs (miR 485-3p, miR-21), and for 2 miRNAs with extraction at 72 h (miR 523, miR 218) (Table 5, raw data S1 Table). Based upon these data using the representative miRNA we have analyzed, plasma extraction less than 12h after phlebotomy appears to provide equivalent results to immediate (within 30 minutes of phlebotomy) extraction.

**Table 5:** Plasma extraction at multiple time points.

	Plasma Extraction Time Comparison			
n = 6	0 vs. 12 hour	0 vs. 24 hour	0 vs. 48 hour	0 vs. 72 hour
miR-374	1.22	-0.01	-0.33	-0.03
p value	0.10	0.98	0.65	0.97
miR-142-3p	0.71	-0.06	-0.26	-0.06
p value	0.24	0.92	0.66	0.92
miR-523	1.23	0.62	-1.35	-2.29
p value	0.24	0.55	0.20	**0.04**
miR-374-5p	0.64	-0.48	-0.19	-0.22
p value	0.22	0.35	0.72	0.67
miR-376c	1.34	0.22	0.19	0.62
p value	0.18	0.82	0.85	0.53
miR-27a	0.49	-0.5	-0.75	-0.23
p value	0.38	0.38	0.19	0.68
miR-520d-5p	1.93	1.68	-0.11	-0.14
p value	0.18	0.26	0.94	0.92
miR-122	-0.04	-2.84	-4.47	-5.94
p value	0.96	**0.00**	**<.0001**	**<.0001**
miR-485-3p	0.51	-0.15	-1.69	-2.22
p value	0.44	0.82	**0.02**	**0.00**
miR-21	0.26	-1.04	-1.48	-1.36
p value	0.69	0.12	**0.03**	**0.05**
miR-218	0.92	0.16	-1.23	-2.56
p value	0.21	0.82	0.10	**0.00**

**Bold** = statistically significant.

### Effect of Repeated Sample Acquisition

Repeated phlebotomy of the same individuals at 12-hour intervals showed no differences in miRNA expression values (Table 6, raw data S2 Table).

**Table 6:** Effect of Repeated Sample Acquisition.

miRNA	ΔΔCT(mean)	p-value
**miR-374**	-0.4458	0.454
**miR-142-3p**	-0.4136	0.4322
**miR-523**	-0.5518	0.3424
**miR-374-5p**	-0.2611	0.6425
**miR-376c**	-0.4312	0.5867
**miR-27a**	-0.3863	0.4776
**miR-520d-5p**	-0.3081	0.7506
**miR-122**	-0.0856	0.9251
**miR-485-3p**	-0.2611	0.6392
**miR-21**	-0.3976	0.5261
**miR-218**	-1.332	0.1659
**miR-374**	-0.4458	0.454

Comparison of mean ΔΔCT’s for various plasma miRNA from plasma samples drawn from the same 7 individuals on the same day 12 hours apart.

### Setting of Cycle Threshold

Setting the cycle threshold bar at a fixed value of 0.03 was preferable to using the default “variable” threshold setting, provided that the number of missing values was less than 10%. Using the modified phenol/guanidine-based lysis and silica membrane-based extraction technique (mirNeasy), only 0.14% of values were missing (see Results‘ section *“Method of RNA Extraction”* below). With this method of extraction, therefore, utilization of a fixed threshold of 0.03 yields reproducible results without the need for normalization required with the use of a variable threshold. The statistical modeling and in depth description of this choice of threshold are discussed elsewhere (Rai et al., unpublished data, 2014).

### Intra-Operator Variability

There was no significant variability between duplicate samples performed by a single operator (Table 7, raw data S3 Table).

**Table 7:** Intra-Operator Variability (Duplicates) for the Trizol LS RNA Extraction and Preamplicification Protocol.

Colorectal Advanced Adenoma + Comparison (n = 32)				
microRNAs	Mean CT Value Duplicate A (±SD)	Mean CT Value Duplicate B (±SD)	|ΔCT|^†^	p-value
miR-374	25.98 (±1.80)	26.00 (±1.93)	0.02	0.9518
miR-142-3p	28.48 (±2.77)	28.74 (±3.05)	0.27	0.6177
miR-523	20.48 (±2.08)	20.21 (±1.71)	0.27	0.4240
miR-374-5p	29.47 (±3.53)	29.81 (±3.61)	0.34	0.6186
miR-376c	32.27 (±2.63)	31.68 (±2.33)	0.58	0.3265
miR-27a	28.62 (±2.25)	29.24 (±2.99)	0.62	0.2216
miR-520d-5p	35.61 (±1.90)	35.24 (±1.77)	0.36	0.4806
miR-122	28.88 (±2.40)	29.11 (±2.82)	0.23	0.6814
miR-485-3p	29.32 (±3.06)	29.44 (±3.32)	0.12	0.8320
miR-21	26.97 (±2.58)	26.96 (±2.81)	0.01	0.9833
miR-218	35.90 (±2.38)	35.83 (±2.08)	0.07	0.9206

^†^ |ΔCT| = absolute value (mean cycle threshold value for duplicate 1 for the miRNA of interest—mean cycle threshold value for duplicate 2 for the miRNA of interest.

### Inter-Operator Variability

Inter-operator variability was assessed using two different miRNA extraction techniques. Inter-operator variability using the Trizol purification and pre-amplification showed one miRNA miR-21 to be significantly different between operators. In addition, a large number of samples were noted to have no miRNA expression for 6 of the 11 evaluated miRNA (Table 8, raw data S3 Table). The large number of samples with no miRNA expression again highlighted the importance of selecting the best extraction technique, since this was not seen with the other method (see also data Table 9). Inter-operator variability using the Qiagen miRNeasy technique yielded 2 miRNAs (miR-485-3p and miR-21) with statistically significantly different ΔCT values (p-values comparing the ΔCT of the two different operators, 0.0191 and 0.0500, respectively) (Table 10, raw data S4 Table). It was believed that these differences in miRNA expression data between operators were within the range of experimental error. Indeed, when these experiments were repeated, these differences were not observed (data not shown). In order to determine whether any inter-observer differences would have a significant impact upon observed group differences we utilized an ANOVA model to examine variations in both miR-21 and RNU6. The group effect remained significant, even when there was an effect of inter-operator variability. These data are presented in S5 Table). The coefficient of variation for RNU6 was 9.1398, and 8.1211 for miR-21. In addition, we determined the mean and standard deviation of 64 measurements of miR-21 performed in plasma samples of patients with adenoma (mean 26.3 ± 1.8, median 25.9) as well as 64 measurements of miR-21 performed in plasma samples of controls (mean 27.6 ± 3.2, median 28.4).

**Table 8:** Differences in ΔCT between operators with Trizol LS RNA extraction and pre-amplification (inter-operator variability).

	Colorectal Advanced Adenoma (n = 16) + Comparison (n = 16)					
	Unpaired t-test—Cycle Threshold = 0.03					
	Operator 1		Operator 2		p-value^¥^	Mean |ΔΔCT|*(±SD)
microRNAs	Mean ΔCT^†^ (±SD)	# miRNA Not Expressed	Mean ΔCT^†^ (±SD)	# miRNA Not Expressed		
miR-374	-1.95 (±2.53)	0	-1.53 (±2.39)	0	0.4974	1.69 (±1.32)
miR-142-3p	0.84 (±1.99)	0	1.23 (±3.06)	0	0.5478	1.69 (±1.83)
miR-523	-7.01 (±3.11)	0	-7.78 (±2.17)	0	0.2551	1.45 (±1.45)
miR-374-5p	2.02 (±2.59)	2	2.83 (±2.85)	3	0.2577	1.69 (±1.73)
miR-376c	5.83 (±2.21)	11	5.48 (±2.71)	11	0.6490	2.42 (±1.42)
miR-27a	1.25 (±1.46)	1	1.50 (±1.77)	5	0.5580	1.29 (±1.23)
miR-520d-5p	8.57 (±3.39)	14	7.00 (±2.73)	17	0.1587	2.05 (±2.03)
miR-122	2.41 (±4.23)	5	2.21 (±3.98)	8	0.8632	1.82 (±1.86)
miR-485-3p	1.48 (±2.35)	0	1.34 (±2.50)	0	0.8182	1.45 (±1.45)
miR-21	-1.83 (±2.60)	0	0.05 (±2.01)	0	**0.0019**	2.40 (±1.82)
miR-218	8.01 (±2.88)	13	8.93 (±2.62)	19	0.3650	1.77 (±1.79)
					Total Average	1.79 (±1.63)

^†^ ΔCT = miRNA of interest cycle threshold value—U6 cycle threshold value.

* |ΔΔCT| = Absolute Value [Operator 1 ΔCT—Operator 2 ΔCT].

^¥^ = p value of mean ΔCT operator 1 vs operator 2.

**Bold** = statistically significant.

**Table 9:** Inter-operator variability with two different extraction methods.

	Trizol LS with Preamplification		Qiagen miRNeasy w/o Preamplification		p-value
microRNAs	Mean |ΔΔCT|*(±SD)	# miRNA Not Expressed	Mean |ΔΔCT|*(±SD)	# miRNA Not Expressed	
miR-374	1.69 (±1.32)	0	0.70 (±0.78)	0	**0.0006**
miR-142-3p	1.69 (±1.83)	0	0.91 (±0.89)	0	**0.0340**
miR-523	1.45 (±1.45)	0	1.38 (±1.02)	0	0.8240
miR-374-5p	1.69 (±1.73)	5	1.27 (±1.06)	0	0.2461
miR-376c	2.42 (±1.42)	22	0.76 (±0.91)	0	**<0.0001**
miR-27a	1.29 (±1.23)	6	0.98 (±1.03)	0	0.2786
miR-520d-5p	2.05 (±2.03)	31	1.20 (±1.01)	1	**0.0405**
miR-122	1.82 (±1.86)	13	1.11 (±1.10)	0	0.0642
miR-485-3p	1.45 (±1.45)	0	1.50 (±1.01)	0	0.8734
miR-21	2.40 (±1.82)	0	1.36 (±1.00)	0	**0.0062**
miR-218	1.77 (±1.79)	32	1.09 (±1.06)	0	0.0692
Total	1.79 (±1.63)	109/704 = 15%^a^	1.12 (±0.99)	1/704 = 0.14%^a^	**<0.0001**
p-value for Number of miRNAs Not Expressed^a^					**<0.0001**

Differences in ΔΔCT between Qiagen miRNeasy with RNA carrier and no pre-amplification and Trizol LS with pre-amplification.

* |ΔΔCT| = Absolute Value [Operator 1 ΔCT—Operator 2 ΔCT].

**Bold** = statistically significant.

^a^ = chi-squared test comparing number of miRNA not expressed using Trizol LS with preamplification as compared to using Qiagen miRNeasy without preamplification.

**Table 10:** Differences in ΔCT between operators with Qiagen miRNeasy with RNA Carrier and No Pre-amplification (Inter-Operator Variability).

	Colorectal Advanced Adenoma (n = 16) + Comparison (n = 16)					
	Unpaired t-test—Cycle Threshold = 0.03					
	Operator 1		Operator 2		p-value^¥^	Mean |ΔΔCT|*(±SD)
microRNAs	Mean ΔCT^†^ (±SD)	# miRNA Not Expressed	Mean ΔCT^†^ (±SD)	# miRNA Not Expressed		
miR-374	-1.80 (±1.75)	0	-1.69 (±1.99)	0	0.8151	0.70 (±0.78)
miR-142-3p	-7.16 (±1.57)	0	-6.60 (±1.81)	0	0.1910	0.91 (±0.89)
miR-523	-3.21 (±2.82)	0	-2.12 (±2.92)	0	0.1339	1.38 (±1.02)
miR-374-5p	-3.27 (±1.94)	0	-2.33 (±1.87)	0	0.0529	1.27 (±1.06)
miR-376c	-0.04 (±1.74)	0	0.05 (±1.98)	0	0.8475	0.76 (±0.91)
miR-27a	-6.12 (±1.56)	0	-5.52 (±1.79)	0	0.1579	0.98 (±1.03)
miR-520d-5p	5.54 (±2.54)	0	6.27 (±2.91)	1	0.2925	1.20 (±1.01)
miR-122	-2.69 (±2.96)	0	-2.05 (±3.58)	0	0.4387	1.11 (±1.10)
miR-485-3p	-2.60 (±2.02)	0	-1.31 (±2.26)	0	**0.0191**	1.50 (±1.01)
miR-21	-7.59 (±1.75)	0	-6.69 (±1.85)	0	**0.0500**	1.36 (±1.00)
miR-218	-3.73 (±2.28)	0	-2.90 (±2.23)	0	0.1460	1.09 (±1.06)
					Total Average	1.12 (±0.99)

^†^ ΔCT = miRNA of interest cycle threshold value—U6 cycle threshold value.

* |ΔΔCT| = Absolute Value [Operator 1 ΔCT—Operator 2 ΔCT].

^¥^ = p value of mean ΔCT operator 1 vs operator 2.

**Bold** = statistically significant.

### Method of RNA Extraction

**Fig 4:**
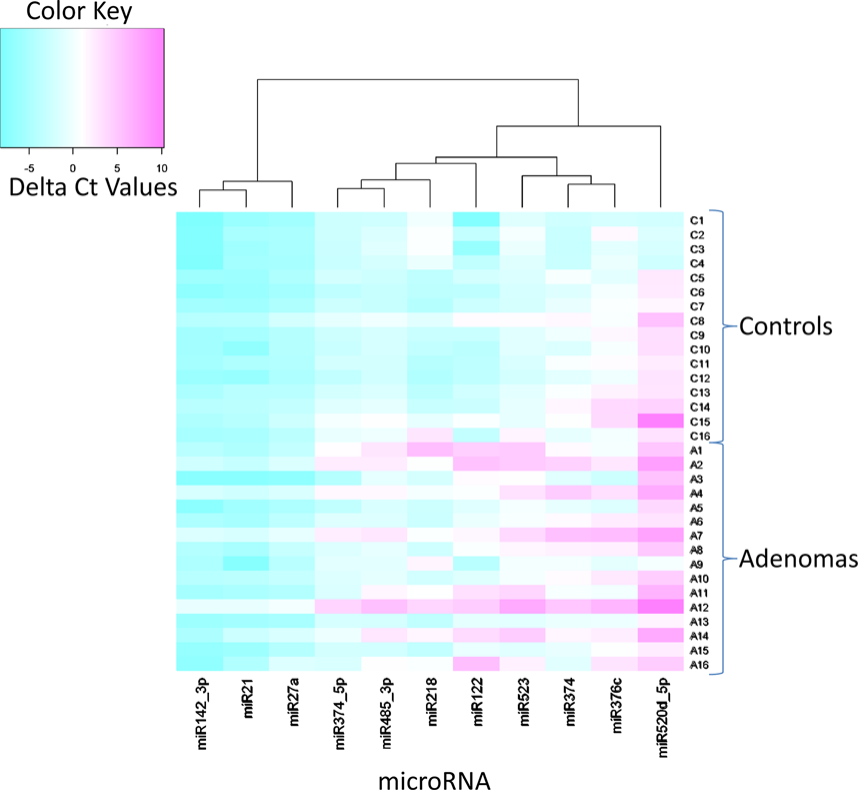
Heat map showing expression of 11 miRNA in plasma of 16 patients with colorectal adenoma prior to treatment and in plasma of 16 controls. Color gradation refers to delta Ct values with RNU6 as reference. Negative values represent over-expression of the target miRNA in comparison to the reference miRNA (RNU6).

The Qiagen miRNeasy extraction *without* pre-amplification resulted in a higher yield of RNA than Trizol LS extraction; therefore, pre-amplification was not necessary and could be omitted as a source of possible variation (Table 11). The average ΔCT was lower with mirNeasy, with fewer missing values than with Trizol purification and pre-amplification. Qiagen miRNeasy extraction *without* pre-amplification resulted in only 1of 704 (0.14%) miRNAs not expressing; however, utilizing the Trizol LS extraction with pre-amplification, 109 of 704 (15%) of miRNAs investigated were not expressed (Table 9). A heat map showing miRNA expression for patient groups is shown in Fig 4.

**Table 11:** Total RNA concentration and purity for Trizol LS and Qiagen miRNeasy with RNA yeast carrier.

Total RNA Extraction Method	Number of Samples	Mean Total RNA Concentration (±SD)	Mean Total RNA Amount	Average Purity (±SD) [A260/A280]	p-value*
Trizol LS	32	19 (±12) ng/μL	380 ng	2.03 (±0.72)	**<0.0001**
Qiagen miRNeasy with RNA Yeast Carrier	32	353 (±85) ng/μL	7060 ng	1.95 (±0.13)	**<0.0001**

*p-value for mean total RNA concentration between methods.

## Discussion

With the growing field of miRNA research and the potential use of miRNAs as biomarkers for cancer and other human diseases, the lack of standardization and reproducibility among studies is becoming more apparent. Such variations in methodology can lead to large variability in results reported from one research group to another. In this study, 5 different sources of variation in experimental technique were examined with respect to their effect on miRNA expression data: 1) time of plasma extraction, 2) method of RNA extraction, 3) cycle threshold setting, 4) intra-operator variability and 5) inter-operator variability. Our brief literature review of a 1-year time frame, was not meant to be an exhaustive review, but rather to provide a “snap-shot” of current practices of performing experiments and analyzing data. Our literature review has shown, that many publications focusing on plasma miRNA do not carefully describe their experimental methods, that there is wide variation in performing such studies and analyzing resulting data.

There has been discussion as to whether plasma or serum is a better for study of circulating miRNA. While some report comparable data others report differences between the two sources [99–101]. We chose to focus on plasma, due to the concerns that miRNAs might be released from blood cells into the serum during the coagulation process as suggested by Wang et al [100].

Our data regarding the timing of plasma extraction suggest that plasma needs to be isolated rapidly, within hours after phlebotomy. With blood samples that were stored at 4°C, even with a modest sample size, we observed statistically significant different ΔCTs for select miRNA beginning as early as plasma extraction at 24 h. This is in agreement with prior reports linking hemolysis to altered miRNA expression [102]. Due to the limited sample size and small number of miRNA investigated, we suggest that plasma be isolated within 12 h following phlebotomy in order to avoid falsely elevated or reduced miRNA expression levels.

The three most widely used methods of total RNA extraction from plasma or serum are guanidinium thiocyanate-phenol chloroform-based methods (e.g., Trizol LS, Life Technologies, Carlsbad, CA), phenol with glass fiber filter-based (e.g. miRVANA, Ambion, Life Technologies), and phenol/guanidine with silica membrane based purification (e.g. miRNeasy, Qiagen, Venlo, The Netherlands). Moret et al. [90] compared all three methods and found that a modified phenol/guanidine lysis with silica membrane-based RNA extraction method yielded enhanced quantity, purity, and performance on assays. Although we did not assess the glass-fiber filter-based method here, our data agree with those of Moret et al. [90] and suggest that the Qiagen miRNeasy with yeast carrier isolation method yields a very reproducible result. The high concentration of 353 +/- 85 ng/μL total RNA obtained with this technique as compared to Trizol LS, which resulted in concentration of 19 +/- 12 ng/μL, allows for pre-amplification to be omitted prior to qPCR, omitting yet another source of data variability. In using this technique, we were able to reduce the number of “missing” samples (miRNA that did not express) from 15% to 0.14%.

Analysis of various fixed as well as of variable threshold settings indicated that a fixed Ct setting of 0.03 produced the most reproducible data provided that < 10% of data were missing. This has the significant advantage of not requiring the additional statistical adjustment that is required when a variable threshold is utilized (Rai et al., unpublished data, 2014).

We demonstrated a lack of significant intra-operator variability. In view of this, triplicates are not necessary, and if there are few missing values (<10%), one could even question the need to perform duplicates given the very low ΔΔCT. Inter-operator variation was also low. Using the phenol/guanidine-based lysis and silica membrane-based purification technique resulted in a narrowing of ΔΔCT values between operators as compared to guanidinium thiocyanate-phenol chloroform purification. In fact, when these experiments were repeated, the differences in miRNA expression between operators for miR-21 and miR-485-3p were not seen, leading us to conclude that this was within the range of experimental error and not a significant issue. The technique of robotic automation could potentially be used to reduce such an experimental error; we did not, however, have access to such technology.

## Conclusions

Trizol LS extraction with pre-amplification results in unacceptable inter-operator variability and should not be utilized when analyzing plasma miRNA. A modified miRNeasy extraction method yields negligible inter-operator variation and the lowest number of missing values. For standardization, we recommend utilizing 0.03 as the cycle threshold bar. No significant intra-operator variability was observed. As such, miRNA studies that are restricted to duplicates rather than triplicates result in greater accuracy and cost savings.

While some inter-operator variation was noted, this was least with the modified phenol/guanidine-based lysis and silica membrane-based RNA purification technique. In addition to the sources of variability noted above, there are many others (Table 1). Similar to the QUADAS tool for the quality assessment of diagnostic accuracy studies, we suggest that studies of plasma miRNA should contain the following information: time to plasma extraction, method of RNA extraction, type of miRNA used (total *vs* exosomal), setting of cycle threshold, the type of quantification used, and details of the statistical analysis [103]. Based upon our studies, rapid plasma extraction is essential. A modified phenol/guanidine-based lysis and silica membrane-based purification RNA extraction is preferred due to the extremely low rate of missing values and high RNA yield, allowing the investigator to avoid using pre-amplification, another source of variability. If there is a low number of missing values (<10%), a fixed threshold setting of 0.03 provides the most reliable, consistent data without the need for data normalization. With this setting, there is no significant intra-operator variability, i.e. replicates can be restricted to duplicates, rather than triplicates, resulting in cost and labor savings. The use of plasma miRNA as biomarkers of human disease is evolving and expanding. Data reproducibility is essential prior to clinical application. Standardization of analytic methods and reporting is necessary to permit accurate data comparison and validation.

## Supporting Information

S1 Table Plasma extraction at multiple time points: within 30 minutes after phlebotomy, and at 12, 24, 48, and 72 h post-phlebotomy. (XLSX)

S2 Table Effect of Repeated Sample Acquisition: samples drawn from each of 7 healthy subjects at 6.30 AM and 12 hours later at 6.30 PM on the same day. (XLS)

S3 Table Intra-Operator Variability and Inter-Operator Variability: using the Trizol purification and pre-amplification. (XLS)

S4 Table Inter-Operator Variability: using the Qiagen miRNeasy technique. (XLS)

S5 Table Variability of miR21 and the housekeeping RNU6. (XLSX)
